# Congenital hypothyroidism: insights into pathogenesis and treatment

**DOI:** 10.1186/s13633-017-0051-0

**Published:** 2017-10-02

**Authors:** Christine E. Cherella, Ari J. Wassner

**Affiliations:** Division of Endocrinology, Boston Children’s Hospital, Harvard Medical School, 300 Longwood Avenue, Boston, MA 02115 USA

**Keywords:** Congenital hypothyroidism, Genetics, Central hypothyroidism, Mild hypothyroidism

## Abstract

Congenital hypothyroidism occurs in approximately 1 in 2000 newborns and can have devastating neurodevelopmental consequences if not detected and treated promptly. While newborn screening has virtually eradicated intellectual disability due to severe congenital hypothyroidism in the developed world, more stringent screening strategies have resulted in increased detection of mild congenital hypothyroidism. Recent studies provide conflicting evidence about the potential neurodevelopmental risks posed by mild congenital hypothyroidism, highlighting the need for additional research to further define what risks these patients face and whether they are likely to benefit from treatment. Moreover, while the apparent incidence of congenital hypothyroidism has increased in recent decades, the underlying cause remains obscure in most cases. However, ongoing research into genetic causes of congenital hypothyroidism continues to shed new light on the development and physiology of the hypothalamic-pituitary-thyroid axis. The identification of *IGSF1* as a cause of central congenital hypothyroidism has uncovered potential new regulatory pathways in both pituitary thyrotropes and gonadotropes, while mounting evidence suggests that a significant proportion of primary congenital hypothyroidism may be caused by combinations of rare genetic variants in multiple genes involved in thyroid development and function. Much remains to be learned about the origins of this common disorder and about the optimal management of less severely-affected infants.

## Background

Thyroid hormone is essential for normal growth and neurologic development, particularly in the first few years of life, and hypothyroidism during this period is a leading cause of preventable intellectual disability worldwide. The implementation of universal newborn screening beginning in the 1970’s has been an enormous public health success, virtually eradicating significant intellectual disability due to severe congenital hypothyroidism in the developed world. Following this early success, newborn screening programs have implemented increasingly stringent screening strategies over the past few decades. The resulting detection of milder cases of congenital hypothyroidism is the primary reason for the dramatic increase in the apparent incidence of congenital hypothyroidism from 1:4000 to 1:2000 newborns over the last 20–30 years [1–6]. However, unlike severe congenital hypothyroidism, for which the benefits of early detection and treatment are indisputable, uncertainty remains about mild disease in terms of the neurodevelopmental risk it poses and whether these risks are mitigated by treatment [7]. Moreover, despite the prevalence of congenital hypothyroidism and our success in treating it, what causes most cases remains a mystery. This review discusses important recent developments in congenital hypothyroidism, focusing on our evolving understanding of its genetics, pathophysiology, and outcomes.

### Primary congenital hypothyroidism

**Fig. 1:**
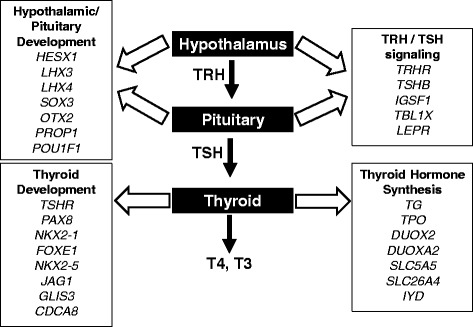
Genes associated with congenital hypothyroidism. TRH, thyrotropin-releasing hormone; TSH, thyroid-stimulating hormone; T4, thyroxine; T3, triiodothyronine

Most congenital hypothyroidism is caused by defects in the thyroid gland itself (primary hypothyroidism). Causes of primary congenital hypothyroidism can be broadly classified as failure of the thyroid gland to develop normally (*dysgenesis*) or failure of a structurally normal thyroid gland to produce normal quantities of thyroid hormone (*dyshormonogenesis*). Thyroid dysgenesis—which encompasses the spectrum of thyroid agenesis, hypoplasia, and ectopy—is the most common cause of congenital hypothyroidism, and its incidence (about 1:4000 infants) has not changed significantly over the last several decades [3, 5, 6]. The underlying cause of thyroid dysgenesis, however, remains obscure in the vast majority of cases. Thyroid dysgenesis usually occurs sporadically, with only 2–5% of cases being attributable to identifiable genetic mutations (Fig. 1). Nevertheless, the known genetic causes of thyroid dysgenesis provide an important window into basic thyroid ontogeny. The thyroid-stimulating hormone receptor (*TSHR*) and the transcription factors *PAX8*, *NKX2–1*, and *FOXE1* are all expressed in the developing thyroid, and disruption of any of these genes can lead to failure of normal thyroid gland formation [8]. These transcription factors also play important roles in other developing tissues, and mutations in each may be associated with additional syndromic features such as renal abnormalities (*PAX8*), interstitial lung disease and chorea (*NKX2–1*), or cleft palate, bifid epiglottis, choanal atresia, and spiky hair (*FOXE1*) (Table 1).

**Table 1:** Clinical features of genetic syndromes associated with congenital hypothyroidism

Primary congenital hypothyroidism		Central congenital hypothyroidism	
*PAX8*	Renal abnormalities	*IGSF1*	Macro-orchidism, delayed pubertal testosterone rise, PRL deficiency, transient GH deficiency
*NKX2–1*	Interstitial lung disease, chorea	*TBL1X*	Hearing deficits
*FOXE1*	Cleft palate, bifid epiglottis, choanal atresia, spiky hair (Bamforth-Lazarus syndrome)	*LEPR*	Severe early-onset obesity, delayed puberty
*NKX2–5*	Congenital heart disease	*POU1F1*	Combined pituitary hormone deficiency
*GLIS3*	Neonatal diabetes mellitus, congenital glaucoma, developmental delay, hepatic fibrosis, polycystic kidneys	*PROP1*	Combined pituitary hormone deficiency
*JAG1*	Alagille syndrome (variable involvement of liver, heart, eye, skeletal, facial defects), congenital heart disease	*HESX1*	Combined pituitary hormone deficiency, optic nerve hypoplasia
*SLC26A4*	Sensorineural hearing loss	*LHX3*	Combined pituitary hormone deficiency, cervical abnormalities, sensorineural deafness
		*LHX4*	Combined pituitary hormone deficiency, cerebellar abnormalities
		*SOX3*	Combined pituitary hormone deficiency, craniofacial abnormalities
		*OTX2*	Combined pituitary hormone deficiency, micro−/anophthalmia, seizures

Several other genes implicated in thyroid dysgenesis offer additional insights into the mechanisms of thyroid development. The transcription factor *NKX2–5* is expressed in the developing heart and thyroid, and *NKX2–5* mutations are associated with congenital cardiac abnormalities. Deletion of *NKX2–5* in mice causes thyroid agenesis, suggesting that this transcription factor plays an important role in thyroid development, but to what degree this finding extends to humans is not clear. Heterozygous variants in *NKX2–5* are found in some individuals with thyroid dysgenesis [9, 10]; however, the pathogenicity of these variants is unclear since they do not consistently cosegregate with thyroid disease in families [9] and some may not impair protein function in vitro [11]. Therefore, the precise role of *NKX2–5* in thyroid dysgenesis remains to be clarified [8].

Mutations in *GLIS3* underlie a complex syndrome of congenital hypothyroidism, neonatal diabetes mellitus, and variable other abnormalities including congenital glaucoma, developmental delay, hepatic fibrosis, and polycystic kidneys [12, 13]. *GLIS3* is highly expressed in the thyroid, and congenital hypothyroidism in patients with *GLIS3* mutations may be associated with either thyroid dysgenesis or a eutopic but histologically abnormal thyroid gland [13]. GLIS3 may act as a transcriptional activator or repressor, but its precise role in thyroid development and function remains to be determined. Some patients with *GLIS3* mutations require unusually high doses of levothyroxine to normalize serum thyroid stimulating hormone (TSH) levels [13, 14], which could imply an additional effect of GLIS3 on central regulation of the hypothalamic-pituitary-thyroid (HPT) axis.

Recently, genetic variants in *CDCA8* (also called *BOREALIN*) were identified in a study of three consanguineous families with thyroid dysgenesis [15]. This gene is expressed in the thyroid and is known to play a key role in the chromosomal passenger complex that stabilizes the mitotic spindle during cell division. Interestingly, however, the *CDCA8* variants detected in these patients do not appear to affect mitosis but rather impair cell migration and adhesion in vitro. Thus, the potential mechanistic role of CDCA8 in thyroid dysgenesis is still unclear, and the range of thyroid phenotypes observed in patients carrying *CDCA8* variants is broad, ranging from thyroid agenesis or ectopy to euthyroid individuals with asymmetric thyroid lobes or thyroid nodules.

While thyroid dysgenesis remains the most common cause of congenital hypothyroidism, the incidence of dyshormonogenesis has been increasing over the last few decades. Whereas dyshormonogenesis accounted for only 15% of congenital hypothyroidism diagnosed in the early days of newborn screening, 30–40% of infants diagnosed by current newborn screening strategies have a eutopic thyroid gland consistent with a form of dyshormonogenesis [3, 5, 6]. [N.B. While the term *dyshormonogenesis* has classically referred to discrete defects in the cellular machinery of thyroid hormone synthesis leading to (often goitrous) congenital hypothyroidism, increasing recognition of the wide spectrum of severity of such defects makes it reasonable to define dyshormonogenesis as inadequate thyroid hormone production from a eutopic thyroid gland].

Unlike thyroid dysgenesis, in which a monogenic cause is present in only a small minority of patients, dyshormonogenesis is frequently due to a genetic defect in some element of thyroid hormone synthesis. Known genetic causes of dyshormonogenesis include mutations in thyroglobulin (*TG*), thyroperoxidase (*TPO*), dual oxidase 2 (*DUOX2*) and its accessory protein (*DUOXA2*), the sodium-iodide symporter (*SLC5A5*), pendrin (*SLC26A4*), and iodotyrosine deiodinase (*IYD*) (Fig. 1). Although dual oxidase 1 (*DUOX1*) is highly homologous to *DUOX2*, isolated defects of *DUOX1* have not been reported to cause congenital hypothyroidism. However, because hypothyroidism due to *DUOX2* mutations tends to be relatively mild, it has been suggested that DUOX1 may partly compensate for DUOX2 deficiency. This hypothesis has been supported by the fact that mice lacking function of both DUOX enzymes have more severe hypothyroidism than those lacking only DUOX2 [16]. More recently, the first evidence of a physiologic role for DUOX1 in humans was provided by a report of two siblings with homozygous inactivating mutations in both *DUOX1* and *DUOX2* associated with congenital hypothyroidism more severe than is typically observed in DUOX2 deficiency alone [17]. While further data are needed, it appears that DUOX1 may indeed serve a redundant role in the human thyroid, not being required for thyroid function under normal circumstances but able to partly compensate when DUOX2 function is impaired.

Despite the growing number of genes associated with congenital hypothyroidism, precisely what proportion of congenital hypothyroidism is attributable to known genetic causes and the relative prevalence of mutations in specific genes are not known precisely, and estimates vary among studies. These variations are influenced by several factors including cohort selection that differs in terms of patient ethnicity and the type(s) of congenital hypothyroidism studied, and the sequencing approaches used to detect mutations. With regard to ethnicity, for example, *DUOX2* appears to be the most commonly implicated gene in East Asian populations, with *DUOX2* variants reported in 16–32% of congenital hypothyroidism patients in Korea, Japan, and China [18–20]. On the other hand, in a cohort of mostly European and Middle Eastern patients, variants in *TG* were much more common (55%) than *DUOX2* variants, which were found in only 18% [21]. However, the latter study was enriched for familial cases of congenital hypothyroidism and is likely to overestimate the prevalence of genetic mutations; therefore, the reported prevalences are likely not generalizable to sporadic cases, which constitute the majority of congenital hypothyroidism seen in clinical practice.

This demographic difference highlights the important influence of cohort selection on the apparent prevalence of genetic mutations in congenital hypothyroidism. Another illustration comes from studies that include patients with congenital hypothyroidism of varying etiologies. For example, one Korean study of 170 infants with congenital hypothyroidism of any etiology found mutations in 31% (most of whom had dyshormonogenesis) [18], while another study from the same country that included only patients with a eutopic thyroid gland identified mutations in 53.5% [22]. Similarly, the prevalence of *DUOX2* variants in Italy has been reported as 15% in unselected patients with congenital hypothyroidism, 23% in those with a eutopic thyroid gland [23], and to 30–37% in those with a eutopic gland and a documented partial iodine organification disorder [24, 25]. Thus, more refined cohort selection can significantly increase the observed prevalence of variants in relevant genes and must be considered when interpreting these data.

Finally, as might be expected, recent studies examining larger sets of candidate genes (often using next-generation sequencing techniques) are increasingly identifying potentially causative variants in a higher proportion of patients than older studies that analyzed only one or a few genes. For example, a recent analysis of 11 genes associated with congenital hypothyroidism in 177 Italian patients with congenital hypothyroidism of any cause demonstrated an overall variant prevalence of 58%; the prevalence was even higher (about 75%) in patients with a eutopic thyroid gland [23]. Many patients (23%) harbored variants in more than one gene, similar to other reports [18, 20, 22, 26]. This consistent finding suggests that the apparent lack of heritability of congenital hypothyroidism may be explained by a confluence of rare variants in several genes. On the other hand, while this hypothesis is intriguing, it remains at odds with the observed high rate of discordance for thyroid dysgenesis among monozygotic twins (who share nearly all variants in all genes) [27], which implies that it is unlikely for a significant proportion of congenital hypothyroidism to be explained by germline genetic changes alone. Another limitation of this and similar genetic studies is that the functional significance of many reported variants—particularly novel missense variants—has not been rigorously evaluated; accordingly, a causal role for these variants in congenital hypothyroidism must be imputed cautiously.

Another novel aspect of this study was to analyze variants in genes associated with both thyroid dysgenesis and dyshormonogenesis in all patients, regardless of their thyroid anatomy. Somewhat unexpectedly, variants in genes typically associated with dysgenesis (e.g., *NKX2–1*, *FOXE1*) were found in patients with dyshormonogenesis, and vice versa [23]. This finding highlights the potential overlap in pathogenesis between the classically distinct phenotypes of thyroid dysgenesis and dyshormonogenesis. An example of such cross-over is *JAG1*, which encodes a ligand of the Notch receptor that is critical for normal thyroid gland formation in zebrafish [28]. Recently, anatomic thyroid defects have been found in a series of patients with heterozygous *JAG1* variants, including both patients with classical Alagille syndrome (a multisystem disorder known to be caused by *JAG1* mutations) and patients with congenital hypothyroidism without syndromic features [29]. These variants were confirmed to disrupt JAG1 function in vivo and strongly support a role for JAG1 in thyroid development in humans. Interestingly, however, the etiologies of hypothyroidism in patients with *JAG1* mutations included not only thyroid dysgenesis, as might be expected from the zebrafish model, but also eutopic thyroid glands. Thus, the case of *JAG1* illustrates the complexity of thyroid development and that the genetic abnormalities underlying the phenotypes of thyroid dysgenesis and dyshormonogenesis may overlap to a greater extent than has been previously appreciated.

### Central congenital hypothyroidism

In contrast to primary disorders of the thyroid gland, central hypothyroidism is caused by dysfunction of hypothalamic or pituitary control of the thyroid axis that leads to inadequate production and/or bioactivity of TSH. Congenital hypothyroidism of central origin is rare: early estimates of its incidence were between 1:29,000 and 1:110,000 [30–32], although more recent data from the Netherlands suggest that it may occur in as many as 1:16,000 newborns and could represent up to 13% of cases of permanent congenital hypothyroidism [33, 34]. Although this incidence is similar to that of phenylketonuria (1:15,000) [35]—the condition for which newborn screening was originally introduced in the 1960’s—central congenital hypothyroidism cannot be detected by the TSH-based screening strategies used by the majority of the newborn screening programs worldwide [1]. Central hypothyroidism may be detected by screening programs that measure T4 concentrations in all infants, along with measurement of TSH either simultaneously or in the subset of infants with low T4. However, this approach may not have optimal sensitivity and may miss some cases of central hypothyroidism [36].

One argument that has been made against routine screening for central hypothyroidism is that it tends to be milder than primary hypothyroidism and is therefore less critical to identify and treat early. Although developmental delays have been reported in small studies of infants who experienced delayed treatment of central congenital hypothyroidism [36, 37], there are no data to demonstrate clearly that early treatment improves outcomes in infants with this condition. However, indirect evidence may be derived from studies of primary congenital hypothyroidism, in which the initial serum concentration of total or free thyroxine (FT4) is one of the most important and consistent predictors of neurodevelopmental outcome [37–40]. In light of this, the premise that central congenital hypothyroidism poses less developmental risk has been challenged by a recent study from the Netherlands demonstrating that 55% of newborns with central hypothyroidism detected on newborn screening had FT4 concentrations sufficiently low (< 10 pmol/L) to warrant treatment according to current consensus guidelines [41, 42]. While few of these patients had the severely low FT4 levels often seen in primary congenital hypothyroidism, their FT4 levels were reduced to a range (5–10 pmol/L) that has been associated with modest deficits at age 10 years [37]. Thus, it appears that a substantial proportion of infants with central congenital hypothyroidism may be at some developmental risk if undetected and untreated, although the precise extent of this risk remains to be determined.

In addition, 75% of infants with central congenital hypothyroidism have additional, potentially life-threatening pituitary hormone deficiencies such as adrenal insufficiency and growth hormone deficiency [34], and detection of these comorbidities represents another argument in favor of screening for central hypothyroidism. Moreover, some have suggested that a carefully designed T4-based screening strategy able to detect these infants may actually be more cost effective than TSH-based screening [33]. In summary, while arguments can be made for routine newborn screening for central hypothyroidism, more compelling evidence is needed to support the need for and feasibility of widespread implementation of such strategies.

Despite its rarity, central congenital hypothyroidism provides an important window into the ontology and physiology of the HPT axis. Normally, thyrotropin-releasing hormone (TRH) from the hypothalamus stimulates thyrotropes in the anterior pituitary to secrete TSH. Congenital defects in this system result from abnormal development of the hypothalamus or pituitary or from genetic alterations that impair the function of TRH or TSH. Developmental or structural anomalies often have broad effects on the hypothalamus and/or pituitary that lead to deficits in multiple pituitary hormones. While some of these cases have no identifiable genetic basis, others can be attributed to mutations in one of several genes critical for the normal early development of these structures, including *HESX1*, *LHX3*, *LHX4*, *SOX3*, and *OTX2* (Fig. 1). These transcription factors have broad effects on fetal development and each is associated with particular syndromic features in addition to combined pituitary hormone deficiency (Table 1). In contrast, the transcription factors *PROP1* and *POU1F1* are expressed later in anterior pituitary differentiation and their disruption results in combined pituitary hormone deficiency without other syndromic features [43].

While central developmental abnormalities often affect multiple pituitary hormones, specific defects in TRH or TSH signaling lead to isolated central congenital hypothyroidism. Until recently the only known genetic causes of this condition were very rare mutations in the TRH receptor (*TRHR*) [44, 45] or the TSH β-subunit (*TSHB*) [26]. However, in 2012 a study of 11 families with central congenital hypothyroidism identified a novel X-linked cause of central hypothyroidism, *IGSF1* [46]. Numerous cases of IGSF1 deficiency have since been described, making it the most common identifiable genetic cause of isolated central congenital hypothyroidism [47].

In addition to central hypothyroidism, males carrying an inactivating mutation of *IGSF1* manifest a clinical syndrome that includes macro-orchidism (88% of patients) and variable hypoprolactinemia (60% of patients). Testicular enlargement can begin before the onset of puberty and has been observed as early as 3 years of age, and affected adults may have testicular volumes up to 45–50 mL. While the normal pubertal increase in testicular size is accelerated in affected individuals, the pubertal rise in testosterone levels appears to be delayed, and plasma testosterone levels remain in the low-normal range in adults. A few children also appear to have transient growth hormone deficiency that resolves by adulthood. Importantly, although the IGSF1 deficiency syndrome is X-linked, 18% of female mutation carriers have central hypothyroidism, about 20% have biochemical prolactin deficiency (although lactation is apparently normal), and up to one-third have late menarche [48, 49].

At the time of its discovery *IGSF1* was known to encode a plasma membrane glycoprotein expressed in anterior pituitary thyrotropes, but its function was unknown. Recently, two studies have begun to elucidate the role of *IGSF1* in the HPT axis and a potential mechanism by which it may cause central hypothyroidism [50, 51]. Both humans and mice deficient in IGSF1 show impaired secretion of TSH in response to exogenous TRH administration, implying a functional defect in TRH signaling. Further studies indicate that IGSF1 directly stimulates TRHR activity in cell culture [50], while Igsf1-deficient mice have reduced pituitary TRHR expression and increased hypothalamic TRH expression [51]. Thus, both in vitro and in vivo evidence suggest that IGSF1 deficiency may cause central hypothyroidism by impairing expression and downstream signaling of the TRH receptor in pituitary thyrotropes. One mechanism by which IGSF1 may promote TRHR signaling is by blocking the inhibitory effect of TGFβ on TRHR expression [50]. Absence of IGSF1 may permit excessive TGFβ-mediated suppression of TRHR that leads to central hypothyroidism. Interestingly, IGSF1 appears to have the opposite effect in pituitary gonadotropes of decreasing FSH β-subunit (*FSHB*) expression. Loss of this inhibition and consequent oversecretion of FSH might explain the macro-orchidism observed in males with IGSF1 deficiency. IGSF1 is also expressed in the Leydig cells and germ cells of the testis, where its role remains uncertain [50]. While more research is needed to understand the mechanisms of IGSF1 action, its discovery has opened the door to the study of novel biology in both the thyroid and gonadal axes.

Recently, mutations in *TBL1X* have been found in several families with X-linked central hypothyroidism [52]. This gene is expressed in the human pituitary and the paraventricular nucleus of the hypothalamus (where TRH-secreting neurons are located), and it encodes a protein that is part of the NCoR-SMRT corepressor complex, a key regulator of thyroid hormone-dependent gene expression. A pathogenic role for TBL1X defects is supported by a mouse model in which impaired NCoR function causes central hypothyroidism [53], and further investigation of the potential role of *TBL1X* in central hypothyroidism is now needed.

### Mild congenital hypothyroidism

As previously noted, most newborn screening programs around the world use TSH-based strategies that effectively detect the vast majority of congenital hypothyroidism [1]. Over the past 30 years, many programs have lowered their screening TSH cut-offs from 20 - 50 mIU/L to 6–15 mIU/L. These changes have resulted in the diagnosis of many more patients with mild congenital hypothyroidism, most of whom have a eutopic thyroid gland [3, 4]. However, in contrast to the known neurodevelopmental risks of severe congenital hypothyroidism and the obvious benefits conferred by its timely and adequate treatment, much less is known about the risks posed by the milder forms of congenital hypothyroidism that are increasingly being diagnosed [7]. This uncertainty is reflected in current consensus guidelines, which find insufficient evidence to recommend for or against the treatment of infants with persistent modest TSH elevation (6–20 mIU/L in serum) but normal levels of FT4 [42]. Therefore, further defining the risks and appropriate treatment of mild congenital hypothyroidism is important, but a randomized, controlled trial to resolve this issue may be difficult to accomplish given the prevailing bias (and perhaps the ethical duty) not to withhold treatment from these infants [42].

Several recent studies have attempted to address this question. A series of studies in Belgian children that assessed the relationship between newborn screening TSH concentrations and various neurodevelopmental outcomes found no relationship between mild TSH elevation (up to 15 mIU/L) and cognitive or psychomotor development or parent-reported behavior scores at 4–6 years of age [54–56]. However, the power of these studies to detect differences in outcomes was limited by the small number of patients with elevated TSH concentrations, particularly in the 10–15 mIU/L range.

A different conclusion was reached by an Australian study that linked newborn screening results with standardized national assessments of childhood development and school performance [57]. This population-based analysis of over 500,000 children found that the risk of poor educational or developmental outcome rose continuously with increasing newborn screening TSH concentration from the 75th to the 99.9th percentile, even after adjusting for potential confounders. Interestingly, no increased risk was observed among infants with screening TSH levels above the 99.9th percentile (12–14 mIU/L), perhaps due to these patients being diagnosed with and treated for congenital hypothyroidism. This study has limitations, including the lack of many patient-level details (including the possibility of diagnosis and treatment of congenital hypothyroidism), inability to account for the potential confounding effect of iodine deficiency, and the inability to establish causality from the observational study design. Nevertheless, the results suggest that mild congenital hypothyroidism may be associated with identifiable neurodevelopmental risks.

Despite the unresolved question of whether infants with mild congenital hypothyroidism benefit from treatment, detecting mild TSH elevations on newborn screening may have other advantages. In particular, a proportion of infants with mild TSH elevation at screening may actually have congenital hypothyroidism that requires treatment. For example, about 12% of infants confirmed to have permanent congenital hypothyroidism—including both dysgenesis and dyshormonogenesis—have only mild TSH elevation at screening [3–5]. Conversely, among infants with mild initial TSH elevation, between 3% and 30% (depending on the specific cut-off used) prove to have permanent congenital hypothyroidism [58, 59]. In a substantial number of these patients, TSH concentrations are much higher when measured in the confirmatory serum sample than was suggested by an initial mild abnormality that would be missed by a higher TSH cut-off [4, 59]. This issue may be particularly significant in preterm and low birth-weight infants with congenital hypothyroidism, in whom the TSH rise may be delayed [60]. Still, these potential advantages of lower TSH cut-offs come at the expense of increased costs of screening, increased parental anxiety over abnormal results of uncertain significance, and the potential for overtreatment with levothyroxine, which itself may be associated with adverse neurodevelopmental outcomes [61]. Thus, in light of currently available data, the true balance of benefits and costs derived from more stringent screening thresholds continues to be debated [62].

## Conclusions

The past 50 years have witnessed extraordinary advancements in the diagnosis, treatment, and outcomes of patients with congenital hypothyroidism. While we still do not understand what causes the majority of congenital hypothyroidism, increasing evidence suggests that a complex interplay of genetic variants in multiple thyroid-related genes may be involved, and the ongoing search for novel genetic causes continues to shed new light on the development and physiology of the hypothalamic-pituitary-thyroid axis. Meanwhile, strong evidence is lacking to guide the management of patients with mild congenital hypothyroidism who have increasingly been diagnosed in recent years. Further high-quality studies are needed to assess the neurodevelopmental risks to these infants and to what extent they may benefit from treatment.

## Abbreviations

FT4: Free thyroxine
HPT: Hypothalamic-pituitary-thyroid
TRH: Thyrotropin-releasing hormone
TRHR: Thyrotropin-releasing hormone receptor
TSH: Thyroid-stimulating hormone
TSHR: Thyroid-stimulating hormone receptor

## References

[1] Ford G, LaFranchi SH (2014). Screening for congenital hypothyroidism: A worldwide view of strategies. Best Pract Res Clin Endocrinol Metab..

[2] Corbetta C, Weber G, Cortinovis F, Calebiro D, Passoni A, Vigone MC, et al. A 7-year experience with low blood TSH cutoff levels for neonatal screening reveals an unsuspected frequency of congenital hypothyroidism (CH). Clin Endocrinol. 2009;71:739–45. 10.1111/j.1365-2265.2009.03568.x 19486019

[3] Deladoey J, Ruel J, Giguere Y, Van Vliet G (2011). Is the incidence of congenital hypothyroidism really increasing? A 20-year retrospective population-based study in quebec. J Clin Endocrinol Metab.

[4] Olivieri A, Corbetta C, Weber G, Vigone MC, Fazzini C, Medda E. Congenital hypothyroidism due to defects of thyroid development and mild increase of TSH at screening: Data from the Italian national registry of infants with congenital hypothyroidism. J Clin Endocrinol Metab. 2013;98:1403–8. 10.1210/jc.2012-3273 23443814

[5] Olivieri A, Fazzini C, Medda E, Collaborators (2015). Multiple factors influencing the incidence of congenital hypothyroidism detected by neonatal screening. Horm Res Paediatr.

[6] Wassner AJ, Brown RS (2015). Congenital hypothyroidism: Recent advances. Curr Opin Endocrinol Diabetes Obes.

[7] Grosse SD, Van Vliet G (2011). Prevention of intellectual disability through screening for congenital hypothyroidism: How much and at what level?. Arch Dis Child.

[8] Szinnai G (2014). Clinical genetics of congenital hypothyroidism. Endocr Dev.

[9] Dentice M, Cordeddu V, Rosica A, Ferrara AM, Santarpia L, Salvatore D, et al. Missense mutation in the transcription factor NKX2-5: A novel molecular event in the pathogenesis of thyroid dysgenesis. J Clin Endocrinol Metab. 2006;91:1428–33. 10.1210/jc.2005-1350 16418214

[10] Wang F, Liu C, Jia X, Liu X, Xu Y, Yan S, et al. Next-generation sequencing of NKX2.1, FOXE1, PAX8, NKX2.5, and TSHR in 100 Chinese patients with congenital hypothyroidism and athyreosis. Clin Chim Acta. 2017;470:36–41. 10.1016/j.cca.2017.04.020 28455095

[11] van Engelen K, Mommersteeg MT, Baars MJ, Lam J, Ilgun A, van Trotsenburg AS, et al. The ambiguous role of NKX2-5 mutations in thyroid dysgenesis. PLoS One. 2012;7:e52685. 10.1371/journal.pone.0052685 PMC3532205 23285148

[12] Senee V, Chelala C, Duchatelet S, Feng D, Blanc H, Cossec JC, et al. Mutations in GLIS3 are responsible for a rare syndrome with neonatal diabetes mellitus and congenital hypothyroidism. Nat Genet. 2006;38:682–7. 10.1038/ng1802 16715098

[13] Dimitri P, Habeb AM, Gurbuz F, Millward A, Wallis S, Moussa K, et al. Expanding the clinical spectrum associated with GLIS3 mutations. J Clin Endocrinol Metab. 2015;100:E1362–9. 10.1210/jc.2015-1827 PMC4596041 26259131

[14] Alghamdi KA, Alsaedi AB, Aljasser A, Altawil A, Kamal NM. Extended clinical features associated with novel GLIS3 mutation: A case report. BMC Endocr Disord. 2017;17:14. 10.1186/s12902-017-0160-z PMC5335837 28253873

[15] Carre A, Stoupa A, Kariyawasam D, Gueriouz M, Ramond C, Monus T, et al. Mutations in BOREALIN cause thyroid dysgenesis. Hum Mol Genet. 2017;26:599–610. 10.1093/hmg/ddw419 PMC6311960 28025328

[16] Grasberger H, De Deken X, Mayo OB, Raad H, Weiss M, Liao XH (2012). Mice deficient in dual oxidase maturation factors are severely hypothyroid. Mol Endocrinol.

[17] Aycan Z, Cangul H, Muzza M, Bas VN, Fugazzola L, Chatterjee VK, et al. Digenic DUOX1 and DUOX2 mutations in cases with congenital hypothyroidism. J Clin Endocrinol Metab. 2017;102(9):3085–90. 10.1210/jc.2017-00529 PMC5587079 28633507

[18] Park KJ, Park HK, Kim YJ, Lee KR, Park JH, Park JH, et al. DUOX2 mutations are frequently associated with congenital hypothyroidism in the Korean population. Ann Lab Med. 2016;36:145–53. 10.3343/alm.2016.36.2.145 PMC4713848 26709262

[19] Matsuo K, Tanahashi Y, Mukai T, Suzuki S, Tajima T, Azuma H, et al. High prevalence of DUOX2 mutations in Japanese patients with permanent congenital hypothyroidism or transient hypothyroidism. J Pediatr Endocrinol Metab. 2016;29:807–12. 10.1515/jpem-2015-0400 27166716

[20] Fan X, Fu C, Shen Y, Li C, Luo S, Li Q (2017). Next-generation sequencing analysis of twelve known causative genes in congenital hypothyroidism. Clin Chim Acta.

[21] Nicholas AK, Serra EG, Cangul H, Alyaarubi S, Ullah I, Schoenmakers E (2016). Comprehensive screening of eight known causative genes in congenital hypothyroidism with gland-in-situ. J Clin Endocrinol Metab.

[22] Jin HY, Heo SH, Kim YM, Kim GH, Choi JH, Lee BH, et al. High frequency of DUOX2 mutations in transient or permanent congenital hypothyroidism with eutopic thyroid glands. Horm Res Paediatr. 2014;82:252–60. 10.1159/000362235 25248169

[23] de Filippis T, Gelmini G, Paraboschi E, Vigone MC, Di Frenna M, Marelli F (2017). A frequent oligogenic involvement in congenital hypothyroidism. Hum Mol Genet.

[24] Rabbiosi S, Vigone MC, Cortinovis F, Zamproni I, Fugazzola L, Persani L (2013). Congenital hypothyroidism with eutopic thyroid gland: Analysis of clinical and biochemical features at diagnosis and after re-evaluation. J Clin Endocrinol Metab.

[25] Muzza M, Rabbiosi S, Vigone MC, Zamproni I, Cirello V, Maffini MA, et al. The clinical and molecular characterization of patients with dyshormonogenic congenital hypothyroidism reveals specific diagnostic clues for DUOX2 defects. J Clin Endocrinol Metab. 2014;99:E544–53. 10.1210/jc.2013-3618 24423310

[26] Nicholas AK, Jaleel S, Lyons G, Schoenmakers E, Dattani MT, Crowne E, et al. Molecular spectrum of TSH-beta subunit gene defects in central hypothyroidism in the UK and Ireland. Clin Endocrinol. 2017;86(3):410-418. 10.1111/cen.13149 PMC5324561 27362444

[27] Perry R, Heinrichs C, Bourdoux P, Khoury K, Szots F, Dussault JH (2002). Discordance of monozygotic twins for thyroid dysgenesis: Implications for screening and for molecular pathophysiology. J Clin Endocrinol Metab.

[28] Porazzi P, Marelli F, Benato F, de Filippis T, Calebiro D, Argenton F (2012). Disruptions of global and jagged1-mediated notch signaling affect thyroid morphogenesis in the zebrafish. Endocrinology.

[29] de Filippis T, Marelli F, Nebbia G, Porazzi P, Corbetta S, Fugazzola L, et al. JAG1 loss-of-function variations as a novel predisposing event in the pathogenesis of congenital thyroid defects. J Clin Endocrinol Metab. 2016;101:861–70. 10.1210/jc.2015-3403 26760175

[30] Fisher DA, Dussault JH, Foley TP, Klein AH, LaFranchi S, Larsen PR (1979). Screening for congenital hypothyroidism: Results of screening one million north american infants. J Pediatr.

[31] Hanna CE, Krainz PL, Skeels MR, Miyahira RS, Sesser DE, LaFranchi SH (1986). Detection of congenital hypopituitary hypothyroidism: Ten-year experience in the northwest regional screening program. J Pediatr.

[32] Persani L (2012). Central hypothyroidism: Pathogenic, diagnostic, and therapeutic challenges. J Clin Endocrinol Metab.

[33] Lanting CI, van Tijn DA, Loeber JG, Vulsma T, de Vijlder JJ, Verkerk PH (2005). Clinical effectiveness and cost-effectiveness of the use of the thyroxine/thyroxine-binding globulin ratio to detect congenital hypothyroidism of thyroidal and central origin in a neonatal screening program. Pediatrics.

[34] van Tijn DA, de Vijlder JJ, Verbeeten B, Verkerk PH, Vulsma T (2005). Neonatal detection of congenital hypothyroidism of central origin. J Clin Endocrinol Metab.

[35] National Institutes of Health Consensus Development P (2001). National institutes of health consensus development conference statement: Phenylketonuria: Screening and management, october 16-18, 2000. Pediatrics.

[36] Nebesio TD, McKenna MP, Nabhan ZM, Eugster EA (2010). Newborn screening results in children with central hypothyroidism. J Pediatr.

[37] Kempers MJ, van der Sluijs Veer L, Nijhuis-van der Sanden RW, Lanting CI, Kooistra L, Wiedijk BM, et al. Neonatal screening for congenital hypothyroidism in the Netherlands: Cognitive and motor outcome at 10 years of age. J Clin Endocrinol Metab. 2007;92:919–24. 10.1210/jc.2006-1538 17164300

[38] Oerbeck B, Sundet K, Kase BF, Heyerdahl S (2003). Congenital hypothyroidism: Influence of disease severity and l-thyroxine treatment on intellectual, motor, and school-associated outcomes in young adults. Pediatrics.

[39] Bongers-Schokking JJ, de Muinck Keizer-Schrama SM (2005). Influence of timing and dose of thyroid hormone replacement on mental, psychomotor, and behavioral development in children with congenital hypothyroidism. J Pediatr.

[40] Kempers MJ, van der Sluijs VL, Nijhuis-van der Sanden MW, Kooistra L, Wiedijk BM, Faber I (2006). Intellectual and motor development of young adults with congenital hypothyroidism diagnosed by neonatal screening. J Clin Endocrinol Metab.

[41] Zwaveling-Soonawala N, van Trotsenburg AS, Verkerk PH (2015). The severity of congenital hypothyroidism of central origin should not be underestimated. J Clin Endocrinol Metab.

[42] Leger J, Olivieri A, Donaldson M, Torresani T, Krude H, van Vliet G, et al. European Society for Paediatric Endocrinology consensus guidelines on screening, diagnosis, and management of congenital hypothyroidism. Horm Res Paediatr. 2014;81:80–103. 10.1159/000358198 24662106

[43] Schoenmakers N, Alatzoglou KS, Chatterjee VK, Dattani MT (2015). Recent advances in central congenital hypothyroidism. J Endocrinol.

[44] Collu R, Tang J, Castagne J, Lagace G, Masson N, Huot C (1997). A novel mechanism for isolated central hypothyroidism: Inactivating mutations in the thyrotropin-releasing hormone receptor gene. J Clin Endocrinol Metab.

[45] Bonomi M, Busnelli M, Beck-Peccoz P, Costanzo D, Antonica F, Dolci C (2009). A family with complete resistance to thyrotropin-releasing hormone. N Engl J Med.

[46] Sun Y, Bak B, Schoenmakers N, van Trotsenburg AS, Oostdijk W, Voshol P, et al. Loss-of-function mutations in IGSF1 cause an X-linked syndrome of central hypothyroidism and testicular enlargement. Nat Genet. 2012;44:1375–81. 10.1038/ng.2453 PMC3511587 23143598

[47] Persani L, Bonomi M (2017). The multiple genetic causes of central hypothyroidism. Best Pract Res Clin Endocrinol Metab.

[48] Joustra SD, Schoenmakers N, Persani L, Campi I, Bonomi M, Radetti G, et al. The IGSF1 deficiency syndrome: Characteristics of male and female patients. J Clin Endocrinol Metab. 2013;98:4942–52. 10.1210/jc.2013-2743 24108313

[49] Joustra SD, Heinen CA, Schoenmakers N, Bonomi M, Ballieux BE, Turgeon MO, et al. IGSF1 deficiency: Lessons from an extensive case series and recommendations for clinical management. J Clin Endocrinol Metab. 2016;101:1627–36. 10.1210/jc.2015-3880 PMC4880178 26840047

[50] Garcia M, Barrio R, Garcia-Lavandeira M, Garcia-Rendueles AR, Escudero A, Diaz-Rodriguez E, et al. The syndrome of central hypothyroidism and macroorchidism: IGSF1 controls TRHR and FSHB expression by differential modulation of pituitary TGF-beta and activin pathways. Sci Rep. 2017;7:42937. 10.1038/srep42937 PMC5338029 28262687

[51] Turgeon MO, Silander TL, Doycheva D, Liao XH, Rigden M, Ongaro L, et al. TRH action is impaired in pituitaries of male Igsf1-deficient mice. Endocrinol. 2017;158:815–30. 10.1210/en.2016-1788 PMC5460797 28324000

[52] Heinen CA, Losekoot M, Sun Y, Watson PJ, Fairall L, Joustra SD, et al. Mutations in TBL1X are associated with central hypothyroidism. J Clin Endocrinol Metab. 2016;101:4564–73. 10.1210/jc.2016-2531 PMC5155687 27603907

[53] Costa-e-Sousa RH, Astapova I, Ye F, Wondisford FE, Hollenberg AN. The thyroid axis is regulated by Ncor1 via its actions in the pituitary. Endocrinol. 2012;153:5049–57. 10.1210/en.2012-1504 PMC3512014 22878400

[54] Trumpff C, De Schepper J, Vanderfaeillie J, Vercruysse N, Van Oyen H, Moreno-Reyes R, et al. Thyroid-stimulating hormone (TSH) concentration at birth in belgian neonates and cognitive development at preschool age. Nutrients. 2015;7:9018–32. 10.3390/nu7115450 PMC4663578 26540070

[55] Trumpff C, De Schepper J, Vanderfaeillie J, Vercruysse N, Van Oyen H, Moreno-Reyes R (2016). Neonatal thyroid-stimulating hormone concentration and psychomotor development at preschool age. Arch Dis Child.

[56] Trumpff C, De Schepper J, Vanderfaeillie J, Vercruysse N, Tafforeau J, Van Oyen H (2016). No association between elevated thyroid-stimulating hormone at birth and parent-reported problem behavior at preschool age. Front Endocrinol (Lausanne).

[57] Lain SJ, Bentley JP, Wiley V, Roberts CL, Jack M, Wilcken B (2016). Association between borderline neonatal thyroid-stimulating hormone concentrations and educational and developmental outcomes: A population-based record-linkage study. Lancet Diabetes Endocrinol.

[58] Langham S, Hindmarsh P, Krywawych S, Peters C (2013). Screening for congenital hypothyroidism: Comparison of borderline screening cut-off points and the effect on the number of children treated with levothyroxine. Eur Thyroid J.

[59] Jones JH, Smith S, Dorrian C, Mason A, Shaikh MG. Permanent congenital hypothyroidism with blood spot thyroid stimulating hormone <10 mu/l. Arch Dis Child. 2016. [Epub ahead of print]. 10.1136/archdischild-2015-309564 27016213

[60] Woo HC, Lizarda A, Tucker R, Mitchell ML, Vohr B, Oh W (2011). Congenital hypothyroidism with a delayed thyroid-stimulating hormone elevation in very premature infants: Incidence and growth and developmental outcomes. J Pediatr.

[61] Bongers-Schokking JJ, Resing WC, de Rijke YB, de Ridder MA, de Muinck Keizer-Schrama SM (2013). Cognitive development in congenital hypothyroidism: Is overtreatment a greater threat than undertreatment?. J Clin Endocrinol Metab.

[62] Lain S, Trumpff C, Grosse SD, Olivieri A, Van Vliet G. Are lower TSH cutoffs in neonatal screening for congenital hypothyroidism warranted? A debate. Eur J Endocrinol. 2017. 10.1530/EJE-17-0107 PMC5763485 28694389

